# Supplementary material for: Nascent dendrite branches initiated by a localized burst of Spire-dependent actin polymerization
Source: Development. 2025 Sep 25;152(18):dev204786. doi: 10.1242/dev.204786 (PMC12516318; doi:10.1242/dev.204786)
Supplement: Supplementary information [file develop-152-204786-s1.pdf]

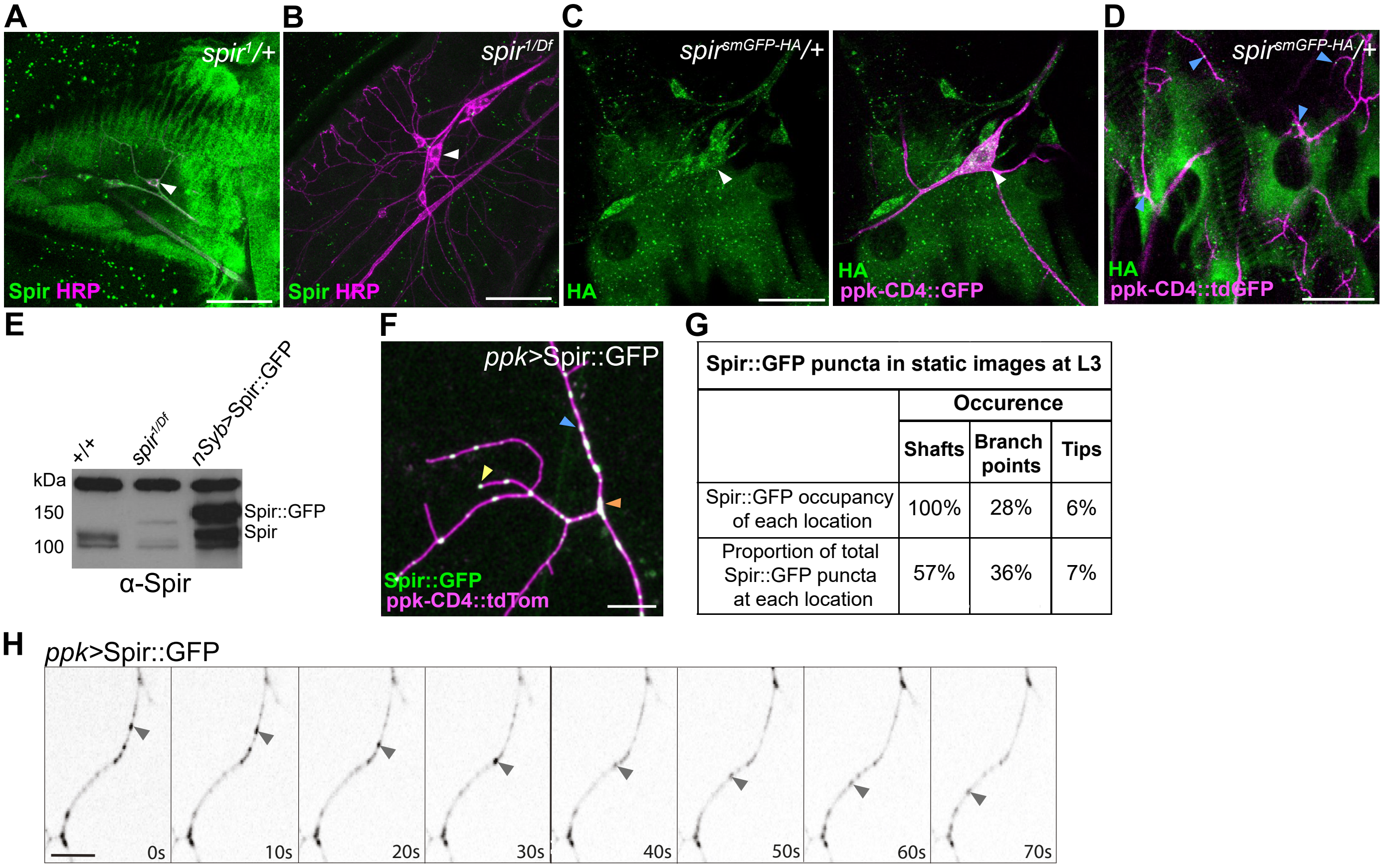

Spir::GFP puncta in static images at L3	Occurence		
	Shafts	Branch points	Tips
Spir::GFP occupancy of each location	100%	28%	6%
Proportion of total Spir::GFP puncta at each location	57%	36%	7%

Fig. S1. Spir expression and motility in dendrites

(related to Fig. 2).

(A, B) IHC co-labeling for endogenous Spir and horseradish peroxidase (HRP) in the body wall of L3 larvae, showing Spir expression in a *spir*^{1}/+ heterozygote (A) but not in a *spir*$^{1/Df}$ hemizygous mutant (B). (C, D) IHC to detect HA-tagged Spir in a Spir-smGFP-HA larva, focusing on cell body of a c4da neuron (C), and distal dendrite arbors (D). (E) Anti-Spir western blot of lysates from dissected adult brains, comparing controls with *spir*$^{1/Df}$ mutants and with flies overexpressing Spir::GFP in all neurons with nSyb-Gal4. Expected mass of Spir::GFP is 142kDa, while that of Spir is 115kDa. The increased presence of Spir (115 kDa) upon overexpression of Spir::GFP likely arises from proteolytic cleavage of the GFP tag. (F) ppk-Gal4 driven Spir::GFP expression is detected with native GFP fluorescence in a c4da neuron labelled with tdTom in an L3 larva. In A-D and F, labelled c4da neuron cell bodies are marked (white arrowheads), as are puncta in dendrite shafts (blue arrowheads in D), branch points (orange arrowhead in F), and tips (yellow arrowhead F). Scale bars in A-D = 25um, F = 10 um. (G) Locations of Spir::GFP puncta observed in static images of c4da dendrite arbors at L3 (n=10 neurons). (H) Greyscale filmstrip (Movie 4) from a c4da neuron of an L2 larva, where the black arrowhead indicates progressive movement of a Spir::GFP-labelled punctum within a dendrite shaft. Scale bar: 5µm. Motile Spir::GFP puncta moved in both anterograde (64%) and retrograde (36%) directions from the cell soma, and sometimes were observed to switch from one to the other. Some puncta appeared to pass one another, while others merged. Motile Spir::GFP particles were found to travel 0.6 um/s on average, and achieved a maximal speed of 1.1 um/s.

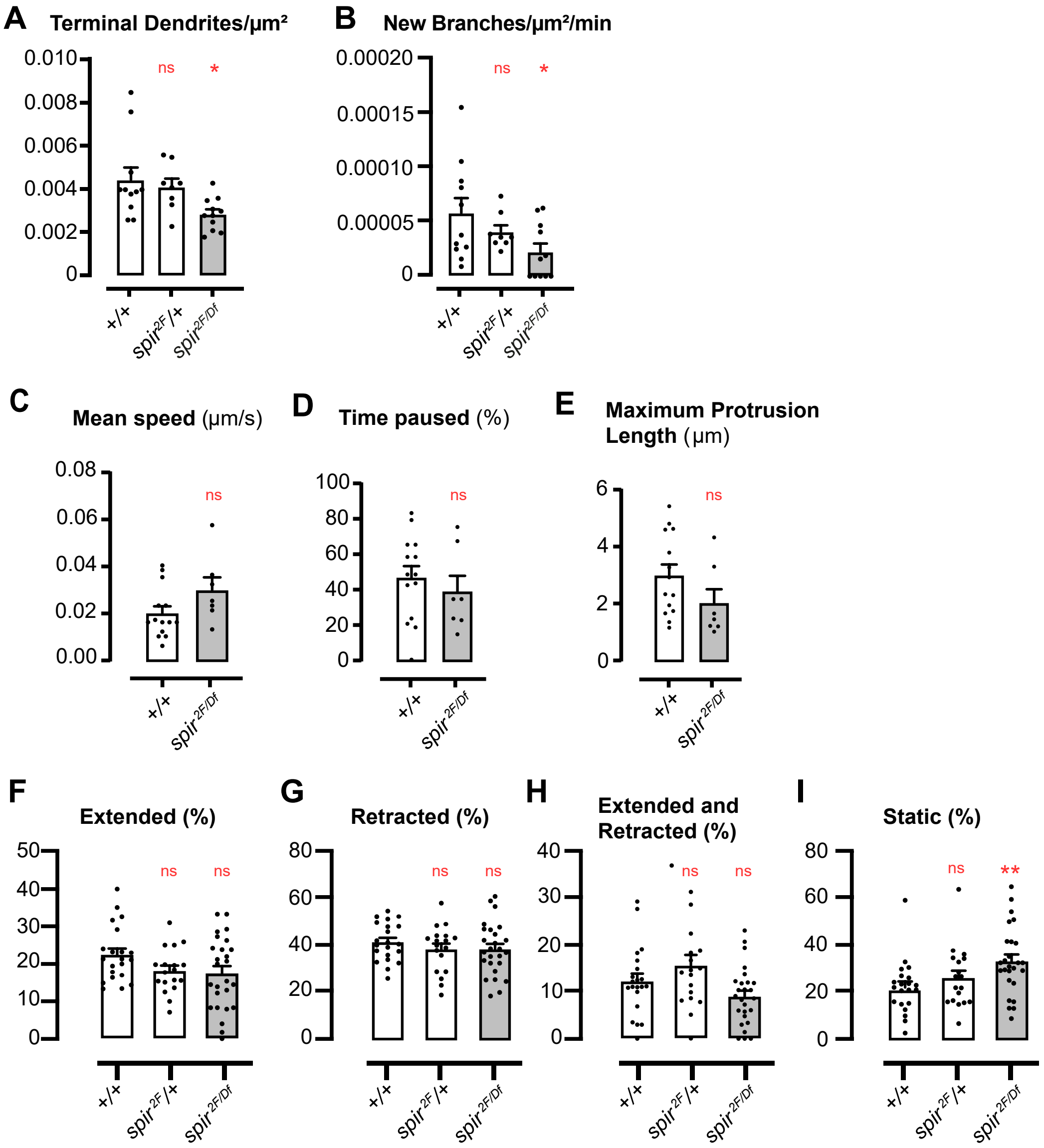

Fig. S2. Quantification of dynamics of newly formed and pre-existing terminal branches

(related to Fig. 2).

(A-B) Dendrite parameters from time-lapse movies of c4da neurons (labeled with ppkCD4::tdTom) in L2 larvae expressing LifeAct::GFP driven by *ppk-GAL4.* Graphs show mean ± SEM, comparing controls (+/+, n=11 movies) with *spir* heterozygotes (*spir*^{2F}/+, n=8) and *spir* mutants (*spir*^{2F}/*spir*^{Df}, n=11). Asterisks indicate significant changes compared to +/+ controls (left-most white bars). (A) Number of terminal branches per µm^2 of area in first frame of each movie (ANOVA ($F(2, 27) = 4.079$, $p = 0.0283$). (B) Number of new branches/µm^2/min in each movie (ANOVA ($F(2, 27) = 3.265$, $p = 0.0537$). (C-E) Protrusive growth characteristics of nascent branches in controls (+/+, n=14 movies) and *spir* mutants (*spir*^{2F}/*spir*^{Df}, n=7) in L2 larvae expressing LifeAct::GFP driven by *ppk-GAL4*, including mean speed (C, t-test ($t(21) = 1.757$, $p = 0.0951$), time paused (D, t-test($t(21) = 0{,}7025$, $p = 0.4909$), and maximum protrusion length (E, t-test ($t(21) = 1.516$, $p = 0.1459$). (F-I) For branches that were already present in the first frame of each movie, quantification of percentage of pre-existing branches that extended (F, ANOVA ($F(2, 62) = 2.482$, $p=0.0919$), retracted (G, ANOVA ($F(2, 62) = 0.6473$, $p=0.5269$), extended and retracted in the same movie (H, ANOVA ($F(2, 62) = 4.058$, $p=0.0221$) or remained static (I, ANOVA ($F(2, 62) = 4.389$, $p=0.0165$). Results in F-I are combined for neurons that expressed LifeAct-GFP and those that did not, since LifeAct-GFP does not affect the number or dynamics of pre-existing branches. Graphs show mean ± SEM, comparing controls (+/+, n=21 movies) with *spir* heterozygotes (*spir*^{2F}/+, n=18) and *spir* mutants (*spir*^{2F}/*spir*^{Df}, n=26).

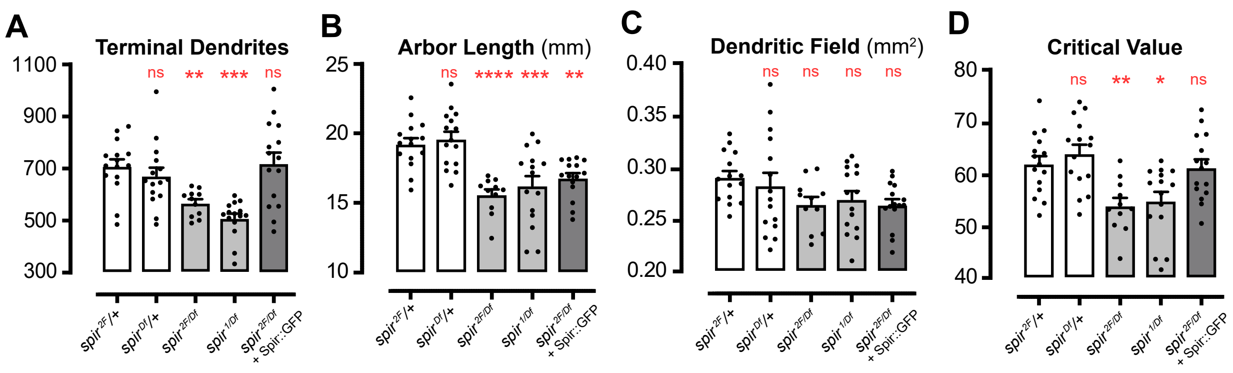

Fig. S3. Dendrite parameters for c4da neurons in L3 larvae

(related to Fig. 3).

(A-D) This is the same dataset shown in Fig. 3, except it was adjusted to control for dendritic field size by removing from the analysis four *spir*$^{2F/Df}$ c4da neurons with unusually small arbors (n=11). Asterisks indicate significant changes compared to *spir*^{2F}/+ heterozygous controls (left-most white bars). (A) Total number of terminal dendrites per neuron (ANOVA ($F(4, 66) = 9.852$, $p < 0.0001$). (B) Total length of dendrite arbor per neuron (mm) (ANOVA ($F(4,66) = 12.03$, $p < 0.0001$). (C) Dendritic field per neuron (mm^2) (ANOVA ($F(4, 66) = 1.912$, $p = 0.1189$. (D) Sholl critical value (ANOVA ($F(4, 66) = 6.914$, $p = 0.0001$).

Development • Supplementary information

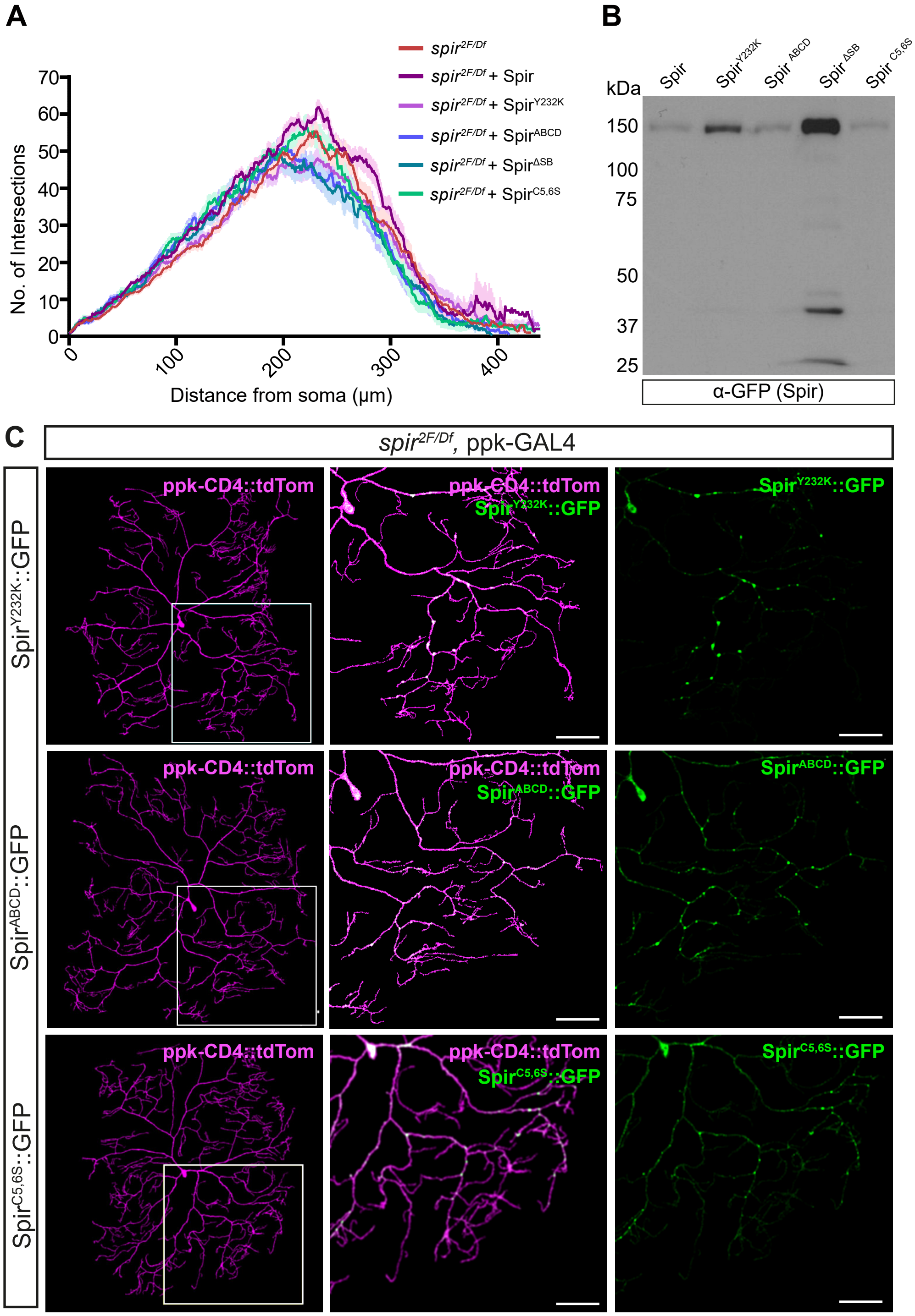

Fig. S4. Function, expression, and distribution of Spir::GFP bearing domain-specific mutations (related to Figure 4).

(A) Sholl profiles for entire c4da neurons labelled with ppk-CD4::tdTom. (B) Anti-GFP western blot from adult heads of animals overexpressing in all neurons either intact Spir::GFP or Spir::GFP bearing domain-specific mutations. One of three replicate blots, this blot is distinct from the one shown in Fig. 4C and is shown in its entirety. The expected molecular weight of Spir::GFP is 142 kDa, and that of GFP is 27 kDa, and so proteolytic cleavage of GFP is not apparent for most constructs. (C) *ppk-GAL4* drives the expression of Spir^{Y232K}::GFP or Spir^{ABCD}::GFP or Spir^{C5,6S}::GFP in a *spir* mutant background. Punctate distribution of Spir::GFP is maintained for each of these mutations. White boxes in left panels indicate areas magnified in panels to their right. Scale bar=50μm.

Development • Supplementary information

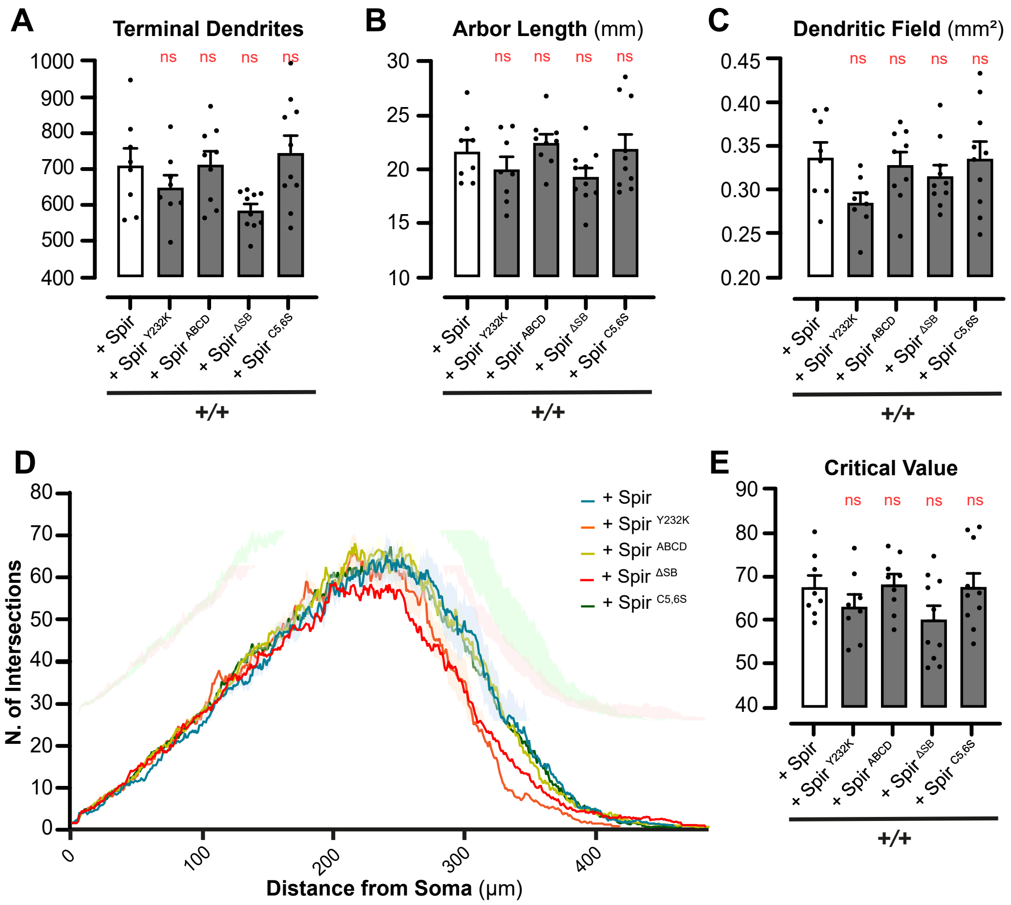

Fig. S5. Effects of overexpression of Spir domain-specific mutations on c4da dendrite arborization in wild-type (+/+) animals
(related to Fig. 4)**.**

(A-E) Dendrite parameters (mean ± SEM) in wild-type L3 larvae (+/+) expressing Spir::GFP ("+ Spir"), or Spir:GFP with domain-specific mutations (n=8-10 for each genotype). There were no significant changes compared to + Spir controls (white bar). All animals in this study bore ppk-Gal4 and ppk-CD4::tdTom transgenes. (A) Total number of terminal dendrites per neuron (ANOVA $F(4, 40) = 3.141$, $p = 0.0245$). (B) Total length of dendrite arbor per neuron (mm) (ANOVA $F(4, 40) = 1.783$, $p = 0.1513$). (C) Dendritic field per neuron (mm^2) (ANOVA $F(4, 40) = 1.843$, $p = 0.1397$). (D) Sholl profiles. (E) Sholl critical value (ANOVA $F(4, 40) = 1.716$, $p = 0.1655$).

Development • Supplementary information

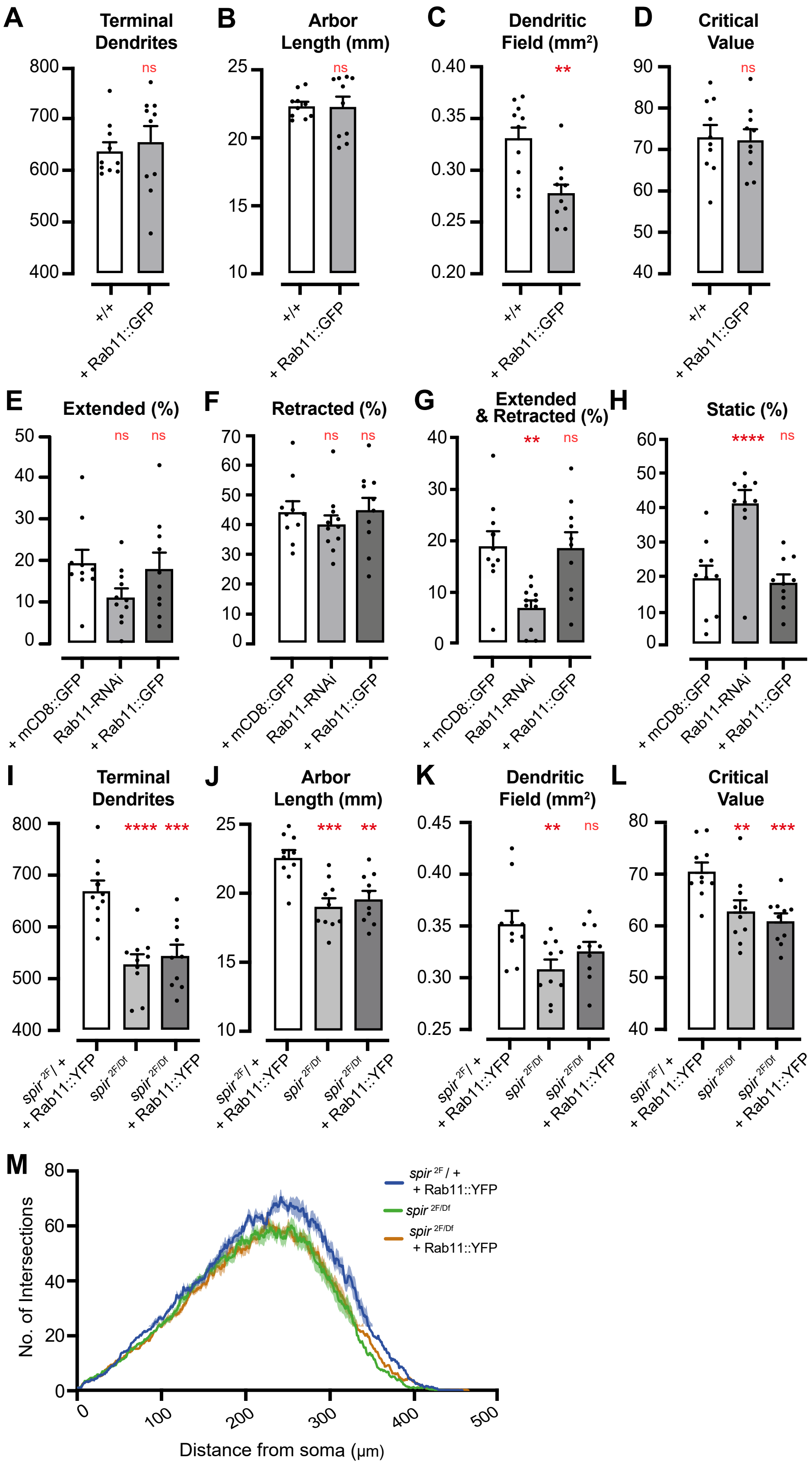

Development • Supplementary information

Fig. S6. Effects of Rab11 on dendrite dynamics at L2 and dendrite arborization at L3 (related to Fig. 5).

(A-D) Dendrite parameters (mean ± SEM) in control L3 larvae (+/+) and ppk-GAL4-driven Rab11::GFP (n=10 for each genotype). Asterisks indicate significant changes compared to indicated controls (white bars). (A) Total number of terminal dendrites per neuron (t-test, $t(18) = 0.5270$, $p = 0.6046$). (B) Total length of dendrite arbor per neuron (mm) (t-test, $t(18) = 0.0571$, $p = 0.9550$). (C) Dendritic field per neuron (mm^2) (t-test, $t(18) = 3{,}551$, $p = 0.0023$). (D) Sholl critical value (t-test, $t(18) = 0.1905$, $p = 0.8510$). (E-H) Dendrite parameters from time-lapse movies of c4da neurons (labeled with ppkCD4::tdTom) in L2 larvae. Graphs show mean ± SEM, comparing controls expressing mCD8::GFP (n=10 movies) with Rab11-RNAi driven by *ppk-GAL4* (n=11) and Rab11::GFP driven by *ppk-GAL4* (n=10). Asterisks indicate significant changes compared to controls (+ mCD8::GFP, white bars). For branches that were already present in the first frame of each movie, quantification of percentage of pre-existing branches that extended (E, ANOVA ($F(2, 28) = 2.301$, $p = 0.1188$), retracted (F, ANOVA ($F(2, 28) = 0.5551$, $p = 0.5802$), extended and retracted in the same movie (G, ANOVA ($F(2, 28) = 8.343$, $p = 0.0014$) or remained static (H, ANOVA ($F(2, 28) = 16.50$, $p<0.0001$). (I-M) L3 dendrite parameters (mean ± SEM) in control *spir*$^{2F/+}$ heterozygotes expressing Rab11::YFP, *spir*$^{2F/Df}$ mutants with or without Rab11::YFP. (n=10 for each genotype). Asterisks indicate significant changes compared to controls (*spir*$^{2F/+}$ heterozygotes + Rab11::YFP, white bars). (I) Total number of terminal dendrites per neuron (ANOVA ($F(2, 27) = 16.21$, $p < 0.0001$). (J) Total length of dendrite arbor per neuron (mm) (ANOVA $F(2, 27) = 12{,}10$, $p = 0.0002$). (K) Dendritic field per neuron (mm^2) (ANOVA ($F(2, 27) = 4.941$, $p = 0.0148$). (L) Sholl critical value (ANOVA ($F(2, 27) = 8.764$, $p = 0.0012$). (M) Sholl profiles.

Figure S7

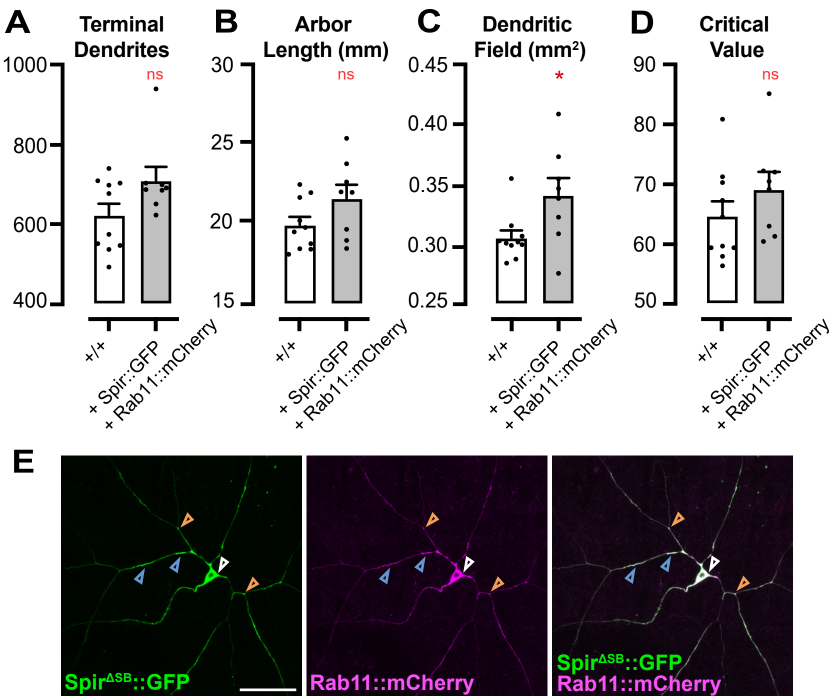

Fig. S7. Effects of co-expression of Spir and Rab11 on dendrite arborization at L3

(related to Fig. 6).

(A-D) Dendrite parameters (mean ± SEM) in control L3 larvae (+/+, n=10) and ppk-GAL4-driven co-expression of Spir::GFP and Rab11::mCherry (n=8). Asterisks indicate significant changes compared to +/+ controls (white bars). (A) Total number of terminal dendrites per neuron (t-test, $t(16)$ =1.963, p = 0.0673). (B) Total length of dendrite arbor per neuron (mm) (t-test, $t(16)$ = 1.767, p = 0.0963). (C) Dendritic field per neuron (mm2) (t-test, $t(16)$ = 2.467 , p = 0.0253). (D) Sholl critical value (t-test, $t(16)$ = 0.1.213, p = 0.2429). (E) *ppk-GAL4*-driven Spir$^{\Delta SB}$::GFP (green) and Rab11::mCherry (magenta). The cell body is shown (white arrowhead), as are examples of co-localization of Spir$^{\Delta SB}$::GFP and Rab11::mCherry at branch points (e.g., orange arrowheads) and dendrite shafts (e.g., blue arrowheads). Scale bar in E=50μm.

Development • Supplementary information

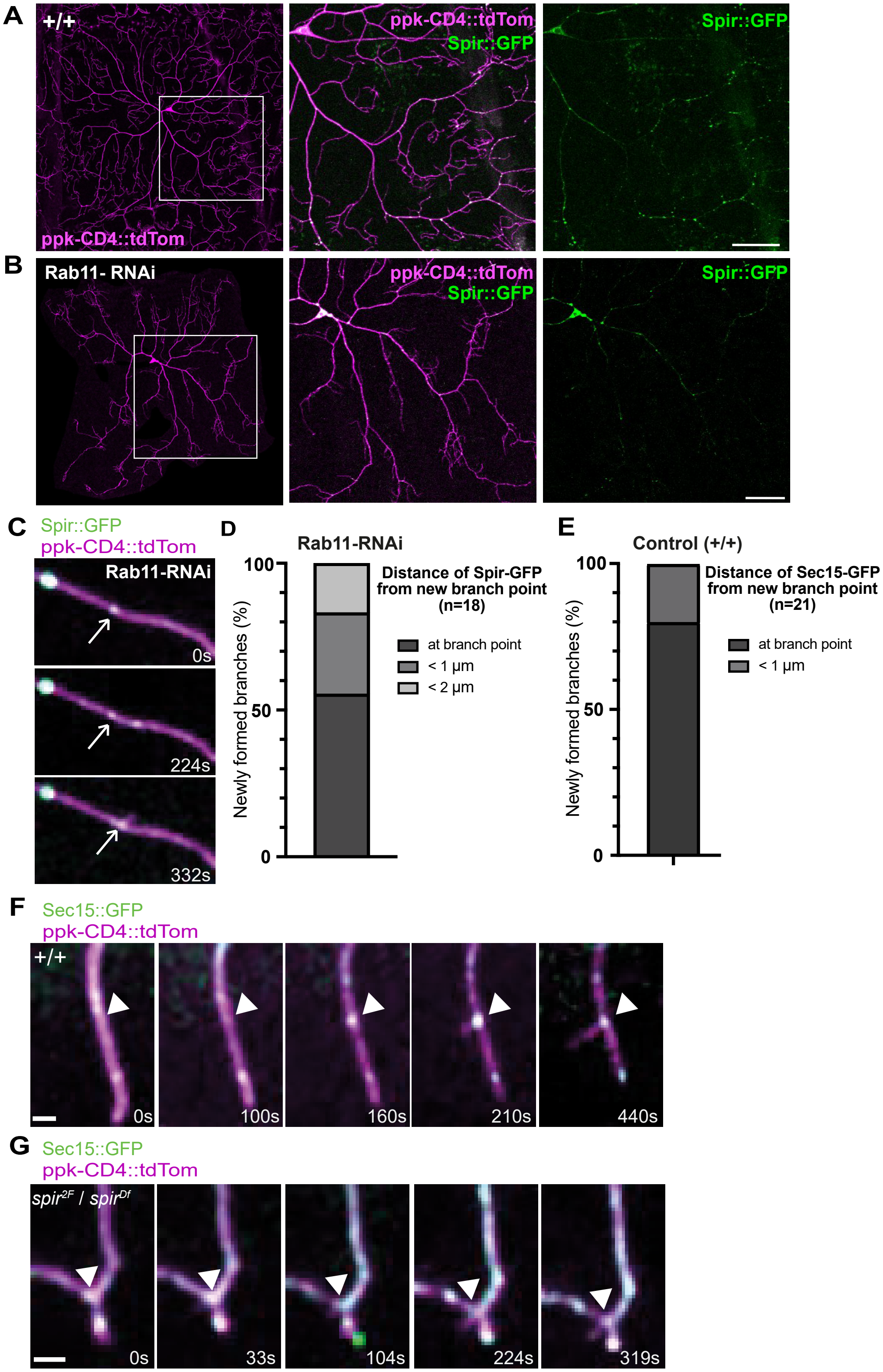
A
+/+
ppk-CD4::tdTom
ppk-CD4::tdTom
Spir::GFP
Spir::GFP
B
Rab11- RNAi
ppk-CD4::tdTom
Spir::GFP
Spir::GFP
C
Spir::GFP
ppk-CD4::tdTom
Rab11-RNAi
0s
224s
332s
D
Rab11-RNAi
Distance of Spir-GFP from new branch point (n=18)
at branch point
< 1 µm
< 2 µm
Newly formed branches (%)
100
50
0
E
Control (+/+)
Distance of Sec15-GFP from new branch point (n=21)
at branch point
< 1 µm
Newly formed branches (%)
100
50
0
F
Sec15::GFP
ppk-CD4::tdTom
+/+
0s
100s
160s
210s
440s
G
Sec15::GFP
ppk-CD4::tdTom
spir2F / spirDf
0s
33s
104s
224s
319s

Fig. S8. Expression, and distribution of Spir::GFP and Sec15::GFP in c4da neurons (related to Figs 5 and 6).

(A, B) Distribution of Spir::GFP in c4da neurons of L3 larvae in controls (+/+, A) and upon Rab11 RNAi knockdown in c4da neurons with *ppk-Gal4* (B). White boxes in left panels indicate areas magnified in panels to their right. Upon knockdown of Rab11 in c4da neurons, Spir::GFP retained punctate distribution within dendrite arbors. Scale bars=50µm. (C) Filmstrip (Movie 8) of Rab11 knockdown L2 larva, where Spir::GFP (arrows) was observed at the initiation site of a new dendrite branch. (D) In Rab11 knockdown c4da neurons, the distance of the closest Spir::GFP puncta from the site of branch initiation (n=18). (E) In control (+/+) c4da neurons, the distance of the closest Sec15::GFP puncta from the site of branch initiation (n=21). (F) Filmstrip (Movie 9) of control (+/+) L2 larva, where Sec15::GFP (arrowhead) pre-exists at the initiation site of a new dendrite branch. (G) Filmstrip (Movie 10) of $spir^{2F/Df}$ mutant L2 larva, with Sec15::GFP (arrowhead) at a rare branch initiation site.

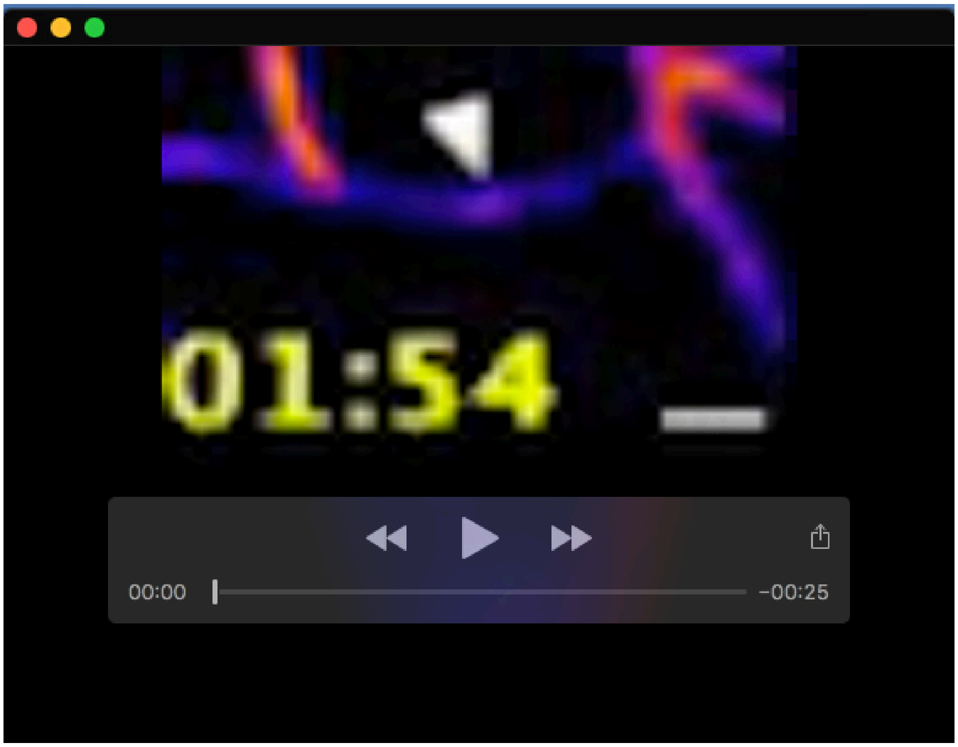

Movie 1. Corresponding to Fig. 1C. 1 frame/7.2s.

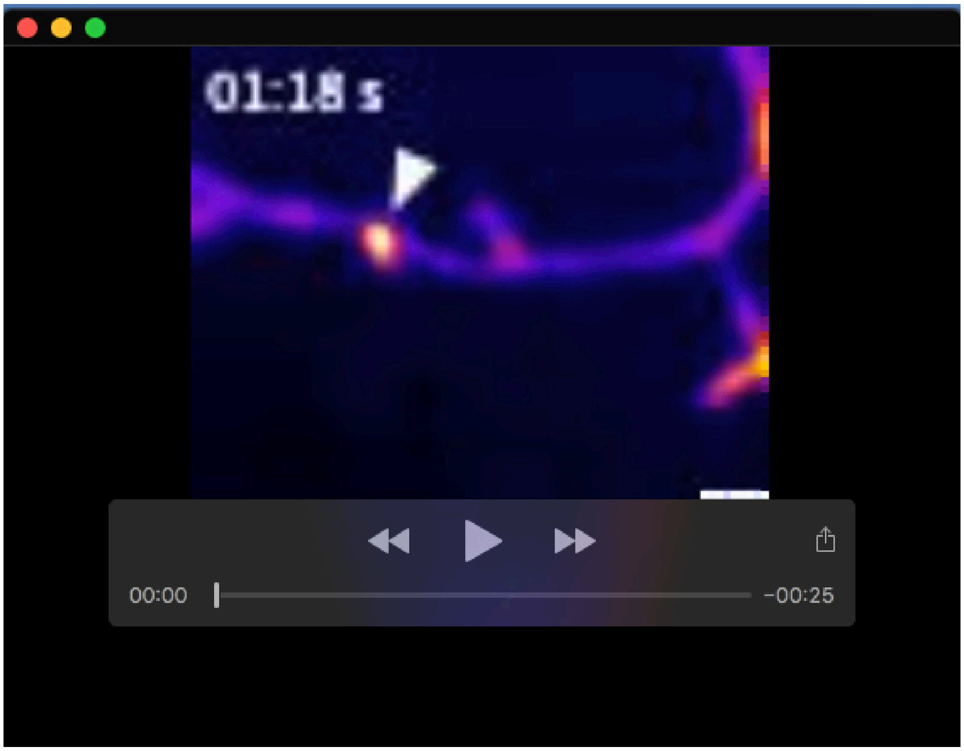

Movie 2. Corresponding to Fig. 1D. 1 frame/4.4s.

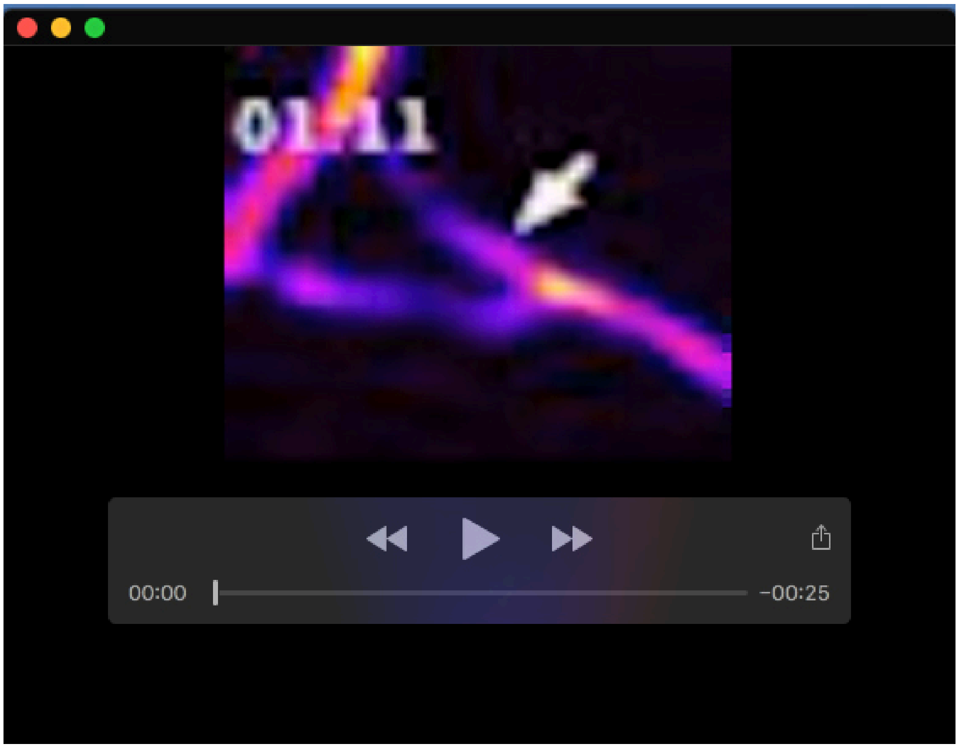

Movie 3. Corresponding to Fig. 1E. 1 frame/6.5s.

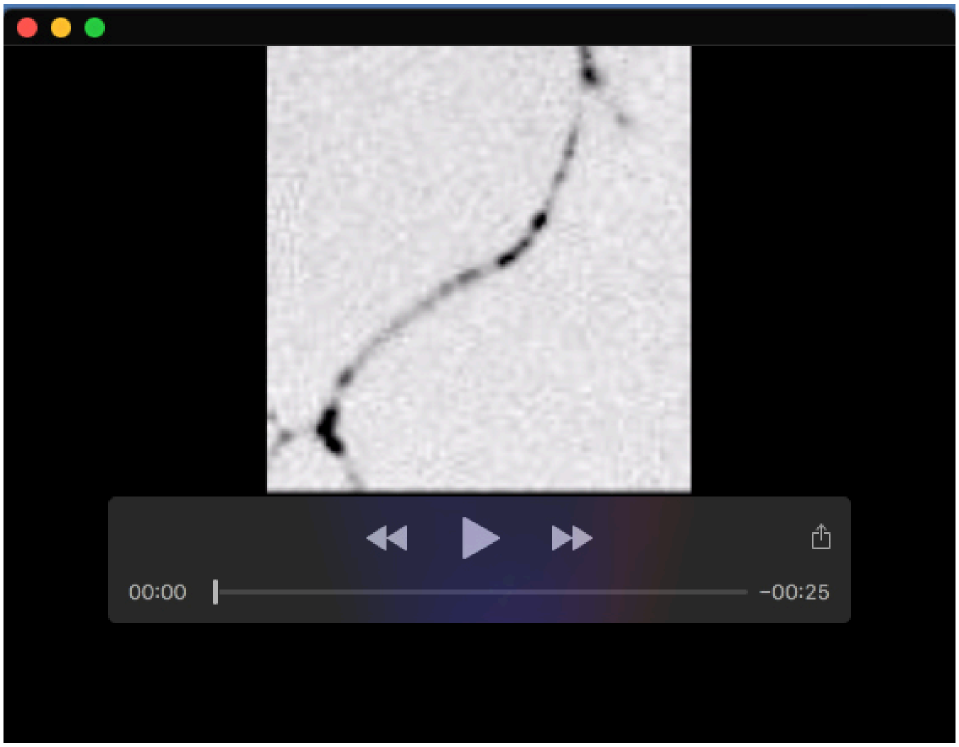

Movie 4. Corresponding to Fig. S1H. 1 frame/~2s.

Development • Supplementary information

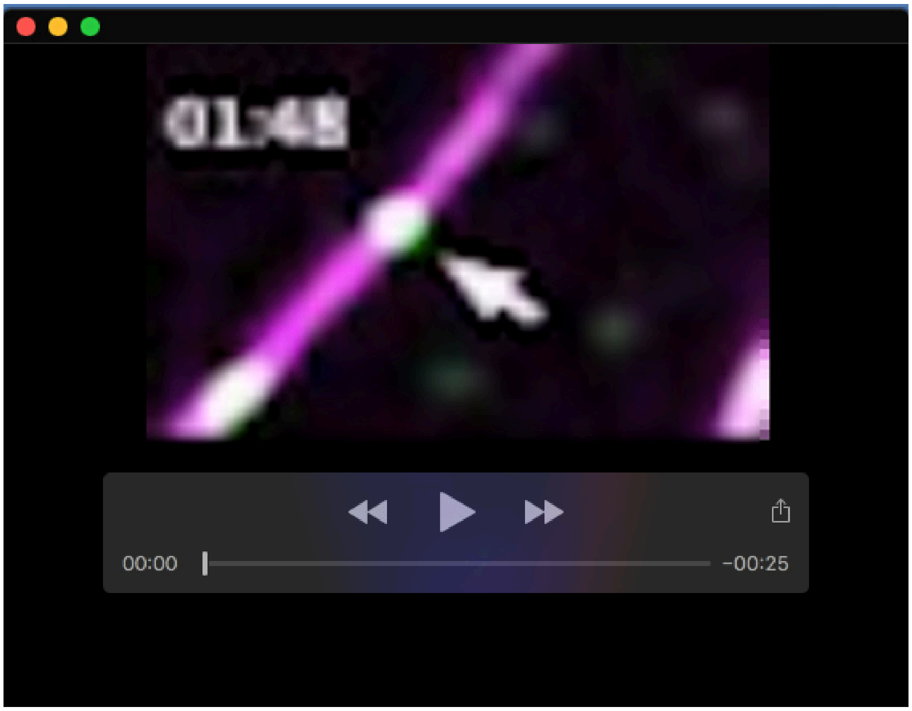

Movie 5. Corresponding to Fig. 2A. 1 frame/~4s.

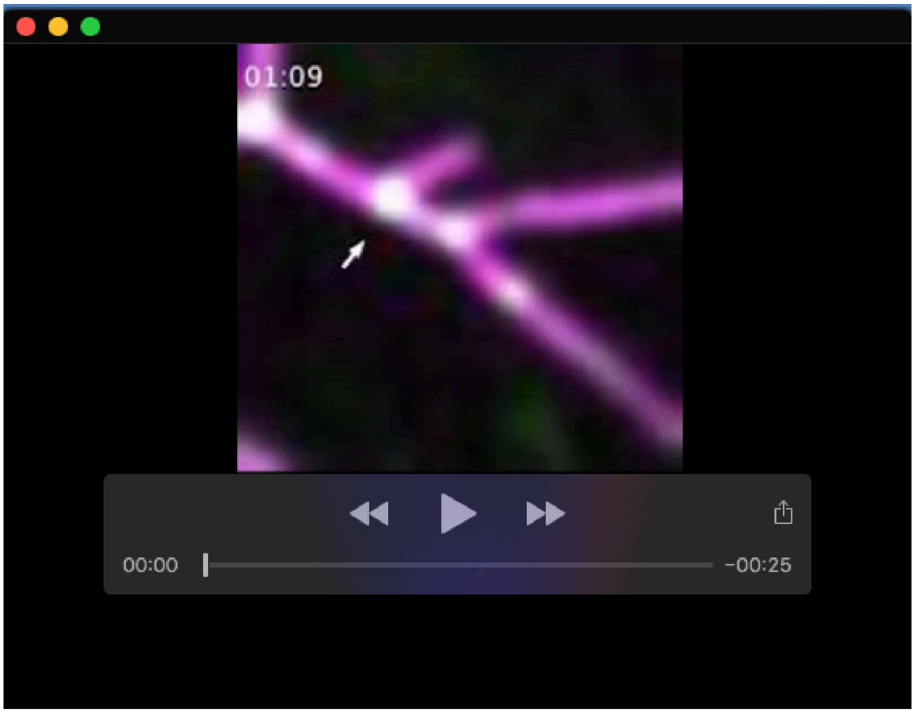

Movie 6. Corresponding to Fig. 2B. 1 frame/~7s.

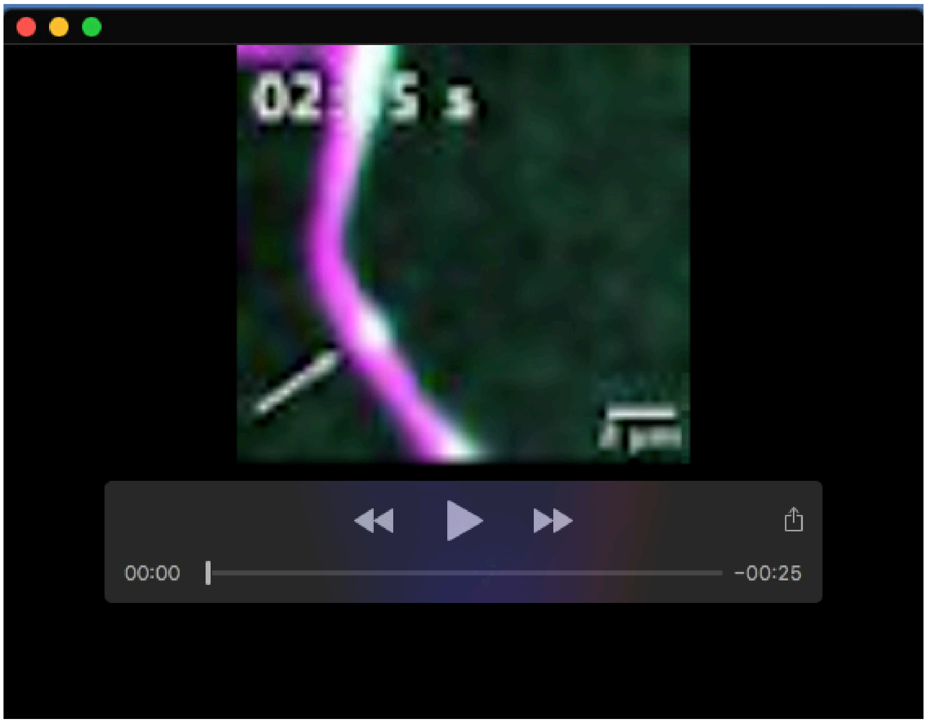

Movie 7. Corresponding to Fig. 5G. 1 frame/~6s.

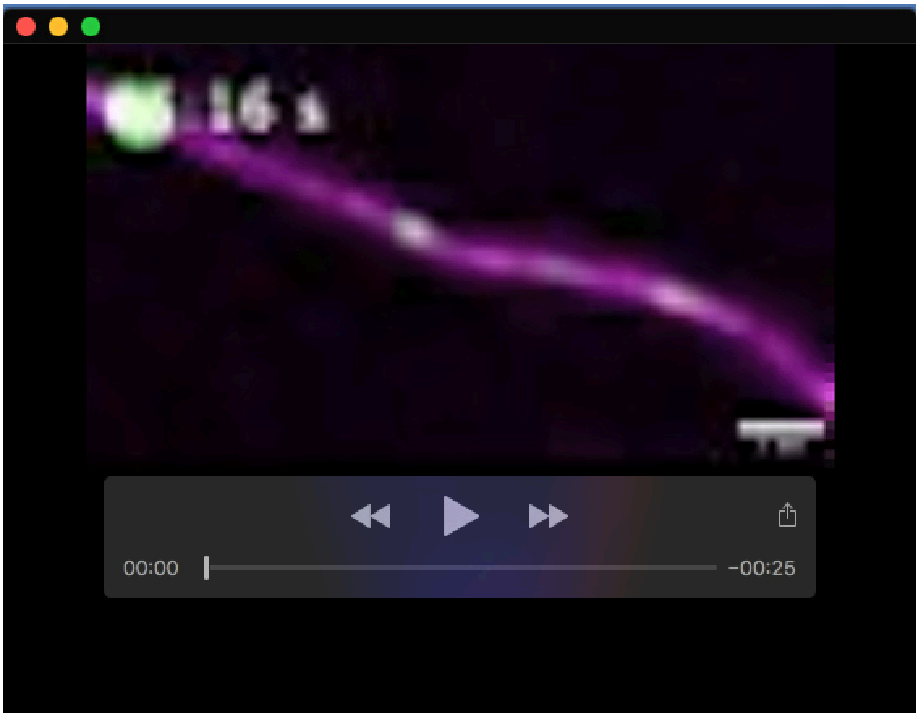

Movie 8. Corresponding to Fig. S8C. 1 frame/~7s

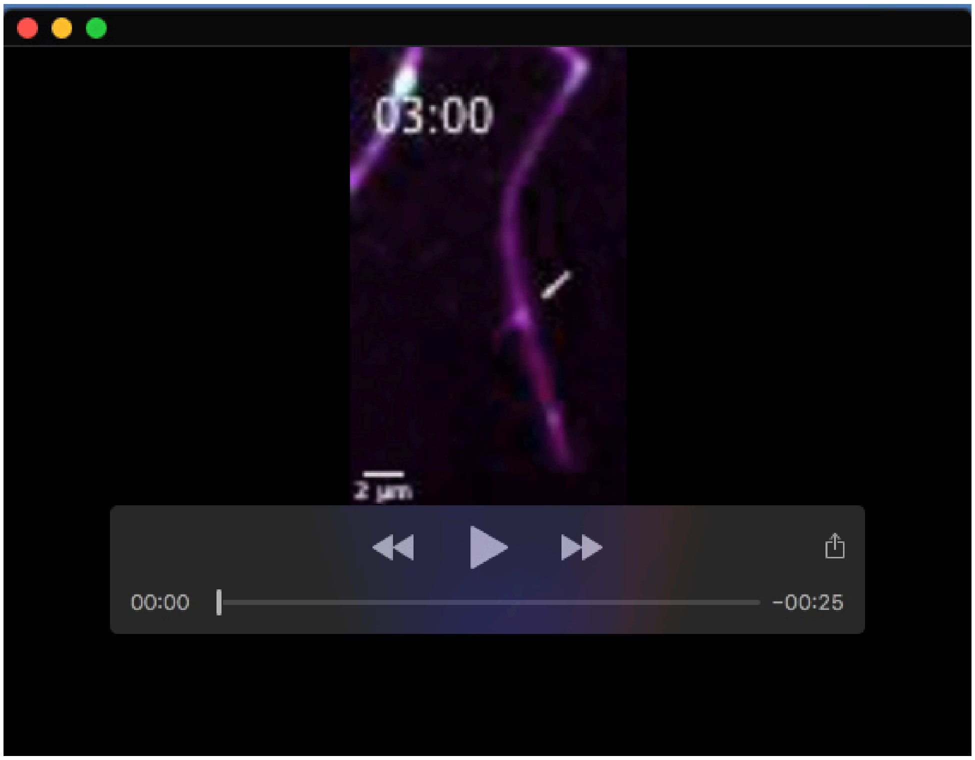

Movie 9. Corresponding to Fig. S8F. 1 frame/~7s.

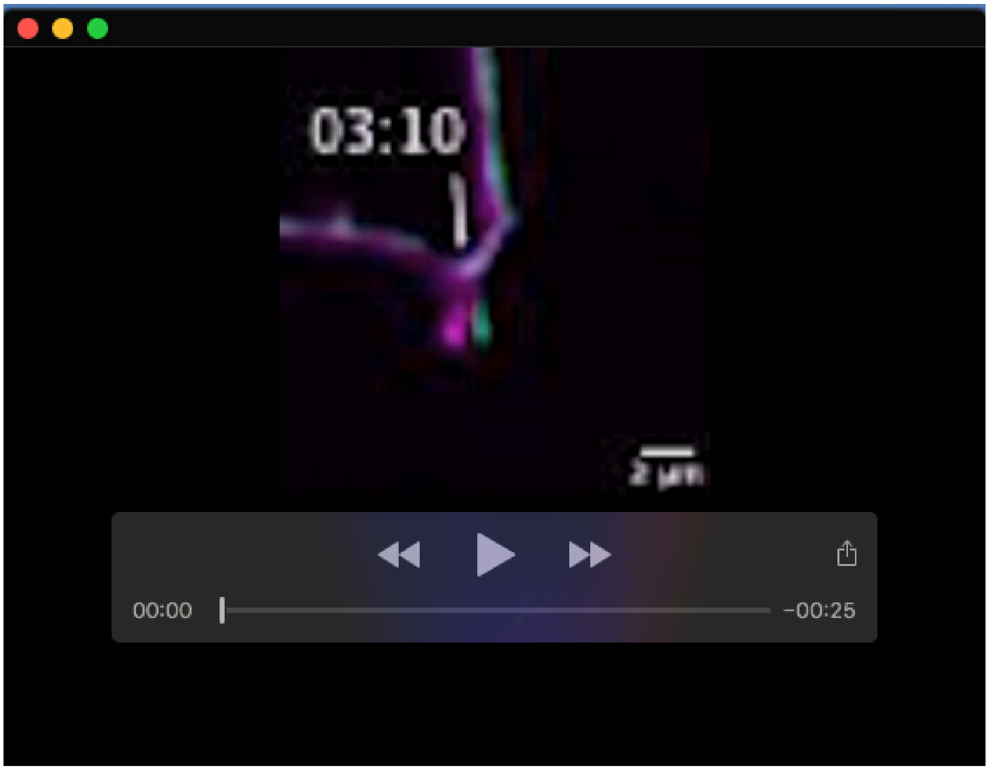

Movie 10. Corresponding to Fig. S8G. 1 frame/~7s.

Development • Supplementary information
